# Therapeutic mucosal vaccination of herpes simplex virus type 2 infected guinea pigs with an adenovirus-based vaccine expressing the ribonucleotide reductase 2 and glycoprotein D induces local tissue-resident CD4+ and CD8+ TRM cells associated with protection against recurrent genital herpes

**DOI:** 10.3389/fimmu.2025.1568258

**Published:** 2025-03-26

**Authors:** Afshana Quadiri, Swayam Prakash, Hawa Vahed, Jimmy Medhat Tadros, Miyo Sun, Kathy K. Hormi-Carver, Swena Jignesh Patel, Lbachir BenMohamed

**Affiliations:** ^1^ Laboratory of Cellular and Molecular Immunology, Gavin Herbert Eye Institute, University of California Irvine, School of Medicine, Irvine, CA, United States; ^2^ Department of Pathology and Laboratory Medicine, School of Medicine, Irvine, CA, United States; ^3^ Institute for Immunology, University of California Irvine, School of Medicine, Irvine, CA, United States; ^4^ Department of Vaccines and Immunotherapies, TechImmune, LLC, University Lab Partners, Irvine, CA, United States

**Keywords:** genital herpes, vaginal mucosa, therapeutic, vaccine, CD4 + T cells, CD8 + T cells

## Abstract

**Introduction:**

The reactivation of herpes simplex virus 2 (HSV-2) from latency causes viral shedding that develops into recurrent genital lesions. The role of tissue-resident T cells and the nature of viral antigens associated with protection against recurrent genital herpes remain to be fully elucidated.

**Methods:**

In this preclinical study, we investigated the protective therapeutic efficacy, in the guinea pig model of recurrent genital herpes, of five recombinant adenovirus-based therapeutic vaccine candidates (rAd-Ags), each expressing different HSV-2 envelope and tegument proteins: RR1 (UL39), RR2 (UL40), gD (glycoprotein D), VP16 (UL48), or VP22 (UL49). We compared the frequency and function of dorsal root ganglia (DRG)- and vaginal mucosa (VM)-resident CD4+ and CD8+ T cells induced by each vaccine and their effect on the frequency and severity of recurrent genital herpes.

**Results:**

HSV-2 latent-infected guinea pigs immunized with rAd-RR2 and rAd-gD vaccines showed high frequencies of DRG- and VM-tissue-resident IFN-g-producing CD4+ and CD8+ TRM cells associated with significant reductions in viral shedding and genital herpetic lesions.

**Discussion:**

Taken together, these preclinical results provide new insights into the T cell mechanisms of protection against recurrent genital herpes and confirm the tegument RR2 protein and glycoprotein D as viable candidate antigens to be incorporated in future genital herpes therapeutic vaccines.

## 1 Introduction

Genital herpes is a recurrent, often painful sexually transmitted disease, caused by herpes simplex viruses type 1 (HSV-1) or type 2 (HSV-2) (1, 2). Most cases of genital herpes are caused by HSV-2. HSV-1 manifests as blisters or cold sores in and around the mouth (3). Herpes simplex virus type 2 (HSV-2) affects both women and men, however, women are more susceptible to the infection (4). Approximately 491 million people are living with HSV type 2 infection in those up to 49 years of age, showing that HSV-2 has a substantial effect on the health of millions of people worldwide (5). After exposure of HSV-2 virus to mucosal genital surfaces, the virus replicates in the mucosal epithelial cells, causing acute genital herpetic lesions. After the initial infection is resolved, the latent virus establishes in the infected ganglia. The virus enters the nerve termini innervating peripheral vaginal tissues and is subsequently transported by retrograde to the nucleus of the sensory neurons of dorsal root ganglia (DRG) where it establishes a dormant state within the neuronal cells (6). The activation of the latent virus causes recurrent disease. The first-in-line treatment of HSV infections is acyclovir, but the recent emergence of ACV-resistant strains emphasizes the need for an effective vaccine against HSV (7–9).

The concept of immunization against viral diseases originated with variolation, the deliberate exposure to small doses of the virulent variola virus. Immunization was markedly improved by the observation that the vaccinia virus produced a mild, localized infection that protected against smallpox. Vaccination is very effective, producing a high level of protection against illness and death that probably lasts a lifetime, although the degree of immunity can wane after 5–10 years (10–12). Interestingly, about herpes, only a small number of vaccine candidates have reached phase 3 trials, and no genital herpes vaccine is FDA-approved despite 75 years of effort (13). Over the last two decades, only a single subunit protein vaccine strategy, based on the HSV-2 glycoprotein D (gD), delivered with or without gB, has been tested and retested in clinical trials. Despite inducing strong neutralizing antibodies, this subunit vaccine strategy proved unsuccessful in clinical trials (14–16). Previous studies have identified other antigenic tegument proteins by screening HSV-2 open-reading frames (ORFs) with antibodies and T cells from HSV-2 seropositive individuals (16). However, aside from three reports, first by our group in 2012 and later by Genocea Biosciences, Inc. in 2014, comparison of the repertoire of HSV-2 proteins, encoded by the over 84 ORFs of the HSV-2 152-kb genome, recognized by antibodies and T cells from HSV-2 seropositive symptomatic versus asymptomatic individuals is largely unknown.

Four main vaccine approaches have been tested in the past four decades to fight herpes simplex virus type 1 (HSV-1) and type 2 (HSV-2) infections and diseases: (1) Inactivated “killed” HSV vaccines; (2) Live-attenuated HSV vaccines; (3) Replication-defective HSV vaccines; and (4) Subunit HSV vaccines. In our previous work, our lab investigated the protective therapeutic efficacy of subunit vaccine candidates that were based on eight recombinantly expressed HSV-2 envelope and tegument proteins in the guinea pig model of recurrent genital herpes. These viral protein antigens (Ags) were rationally selected for their ability to recall strong CD4^+^ and CD8^+^ T-cell responses from naturally “protected” asymptomatic individuals, who, despite being infected, never develop any recurrent herpetic disease. Out of the eight HSV-2 proteins, the envelope glycoprotein D (gD), the tegument protein VP22 (encoded by the UL49 gene), and ribonucleotide reductase subunit 2 protein (RR2; encoded by the UL40 gene) produced significant protection against recurrent genital herpes. Four out of the eight HSV-2 proteins—the tegument protein VP16 (encoded by the UL48 gene), the tegument protein VP11/12 (encoded by the UL46 gene), the tegument protein VP13/14 (encoded by the UL47 gene), and gB (encoded by the UL27 gene)—produced moderate yet significant protection (14). Ribonucleotide reductase (RR) plays a key role in the synthesis of DNA precursors and catalyzes the reduction of the four abundant ribonucleotides into their corresponding limiting deoxyribonucleotides (17). The protein is formed by the association of two non-identical subunits called RR1 and RR2, each consisting of two identical polypeptide chains. The RR1 subunit binds the substrates and the allosteric modulators, provides thiol groups involved in the reduction reaction, and interacts with thioredoxin and/or glutaredoxin for the regeneration of active thiol groups. The R2 subunit contains the cofactor essential for ribose reduction, a tyrosyl radical adjacent to an oxo-bridged dinuclear iron center (18). Glycoprotein D (gD) is a structural component of the herpes simplex virus envelope that is being used as an antigen in various anti-herpes subunit vaccines owing to its involvement in binding the host cell receptors for host infectivity (19, 20). VP22 is a highly abundant tegument protein in HSV with more than 2000 copies in each virion. It is important for the secondary tegumentation of the virion and the accurate localization of several important herpes viral proteins, including the transcription activating protein VP16 (21). It interacts with VP16 and glycoproteins, regulates the assembly of viral particles, and promotes the formation of mature viral particles (20). VP16, as a tegument, is also involved in viral assembly. VP16 is an important transactivator that can activate the transcription of viral immediate-early genes. VP16 and VP22 participate in innate immune evasion (22).

Previously, we demonstrated that the HSV-2 specific RR2 protein-based subunit therapeutic vaccine elicited a significant reduction in virus shedding and decreased the severity and frequency of recurrent genital herpes lesions (16). Additionally, the protein boosted the number and function of antiviral tissue-resident memory CD4^+^ and CD8^+^T_RM_ cells, locally within the DRG and vaginal mucocutaneous tissues, leading to better protection against recurrent herpes (15).

With the advent of COVID-19, adenoviruses are being used as vectors in immunization strategies against viral infections (23, 24). These viral vectors carry genetic material encoding antigens from a target pathogen. When administered to a host, they trigger an immune response against the encoded antigen, thereby priming the immune system to recognize and combat the actual pathogen should an infection occur. In the present study, we evaluated the protective efficacy of five recombinant adenovirus-based therapeutic vaccines against acute and recurrent HSV-2 infection in guinea pigs. Adenovirus vectors were engineered to carry these genes that encode five different antigens from the herpes virus. These five antigens were RR1(UL39), RR2(UL40), gD(glycoprotein D), VP16(UL48), and VP22(UL49) viral antigens, that have been previously tested in our lab for their vaccine potential and have shown to provide moderate to significant protection as subunit vaccines (22). Recombinant adenovirus-based vaccines showed higher reductions in genital lesions and stronger antigen-specific T-cell responses. Moreover, the severity of genital lesions in guinea pigs immunized with Ad-antigens was significantly decreased compared to mock-vaccinated animals. These results suggest that the recombinant adenoviruses have potential as therapeutic vaccine candidates.

In the present study, the rAd-RR2 and rAd-gD vaccines have manifested in being superior or comparable to dl-529 in inducing T-cell responses, IFN-γ response, or reducing viral titers. Moreover, the rAd-RR2 and rAd-gD were also comparable to dl-529 in preventing recurrence, hence posing a potential therapeutic vaccine against recurrent infections. Dl-529 is a replication-defective HSV-2 mutant used as a positive control.

## 2 Results

### 2.1 Therapeutic immunization of HSV-2 infected guinea pigs with Adenovirus-5 expressing RR2 and gD protects against recurrent genital disease

**Figure 1:**
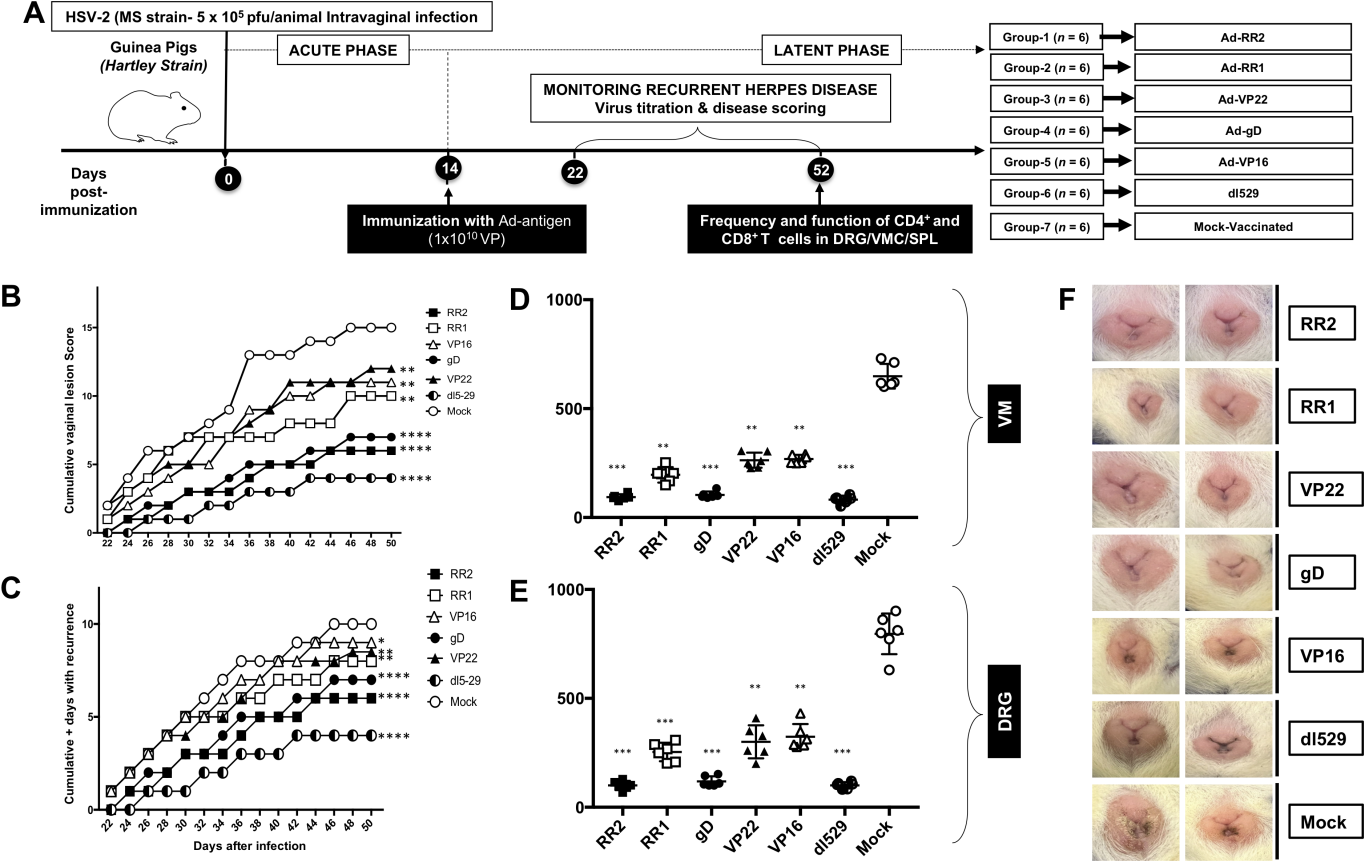
Protection against genital herpes infection in HSV-2 infected guinea pigs following therapeutic immunization with five adenovirus-based vaccine candidates **(A)** Timeline of HSV-2 infection, virological and immunological analysis. Guinea pigs were infected intravaginally on day 0 with 5 X 10^5^ PFU of HSV-2 (MS strain). Once the acute infection was resolved latently infected animals were divided into 7 groups and then immunized intravaginally on day 15 with 10^10^ genomic copies of AD-5 expressing HSV-2 antigens (RR2, RR1, gD, VP22 and Vp16). The replication defective dl529 mutant vaccine was used as a positive control. From day 25 to 56 post-infection, the guinea pigs were monitored daily for the severity of genital herpes lesions, scored on a scale of 0-4 and vaginal swabs were collected to detect virus shedding and to quantify HSV-2 DNA copy numbers. **(B)** Cumulative scoring of vaginal lesions observed during recurrent infection **(C)** Cumulative positive days with recurrent genital lesions. **(D)** HSV-2 DNA copy numbers detected in the VM of vaccinated and mock-vaccinated guinea pig groups. **(E)** HSV-2 DNA copy numbers detected in the DRG of vaccinated and mock-vaccinated guinea pig groups. **(F)** Representative images of genital lesions in guinea pigs vaccinated with Ad-RR2, Ad-RR1, Ad-VP22, Ad-gD, Ad-VP16,dl-529 and Mock. * = P<0.05, ** = P≤0.01, *** = P≤0.001, **** = P≤0.0001.

Guinea pigs (n = 42) were infected intravaginally with 5 x 10^5^ pfu of HSV-2 (strain MS) (**Figure 1A**). Once acute infection was resolved, latently infected animals were randomly divided into seven groups (n = 6) and then vaccinated intravaginally on days 15 post-infection with Ad5 expressing RR2, RR1, gD, Vp22 and VP16 proteins (rAd-RR2, rAd-RR1, rAd-gD, rAd-VP22, and rAd-VP16). Dl529 treated animals were used as positive control while mock-vaccinated guinea pigs (n = 6), that received adenovirus alone, were used as negative controls. From day 22 until day 52, the guinea pigs were observed and scored regularly for genital lesions. The vaccinated animals exhibited significantly lower cumulative vaginal lesions (**Figure 1B**) and an overall significant reduction in cumulative positive days of recurrence compared to the mock-vaccinated controls (**Figure 1C**). rAd-RR2, and rAd-gD vaccinated animals displayed lowest cumulative vaginal lesions and an overall reduced cumulative positive days of recurrence compared to the other vaccinated animals (**Figures 1B, C**).

On day 52 post-infection, the guinea pigs were sacrificed to quantify the amount of HSV-2 challenge virus in their DRG (Dorsal Root Ganglia) and VM (Vaginal Mucocutaneous Tissue) using PCR. We observed that the vaccinated guinea pigs exhibited lower HSV-2 DNA copy numbers in the DRG and VM than did mock-vaccinated controls (**Figures 1D, E**), which was associated with a significant reduction in cumulative virus vaginal shedding in the vaccinated group as compared to the mock-vaccinated control group. rAd-RR2, and rAd-gD vaccinated animals displayed lowest HSV-2 DNA copy numbers in the DRG and VM, which was comparable to dl529 (**Figures 1D, E**). The severity of genital herpetic lesions scored on a scale of 0 to 4, also confirmed the cure of recurrent disease in rAd-RR2, and, rAd-gD vaccinated guinea pigs (**Figure 1F**). The lowest genital lesions were observed in guinea pigs vaccinated with rAd-gD and rAd-RR2 (**Figure 1F**). The other vaccinated groups were moderately protective against genital lesions (**Figure 1F**). However, the mock- vaccinated group showed no significant protection against recurrent genital herpes lesions (**Figure 1F**, *bottom panel*). Altogether, these results indicate that therapeutic immunization with AD-RR2 and AD-gD protected HSV-2-infected guinea pigs against recurrent genital herpes infection and disease.

### 2.2 Therapeutic immunization of HSV-2 infected guinea pigs with Ad5-antigen produced a higher antibody titer against the corresponding antigen

**Figure 2:**
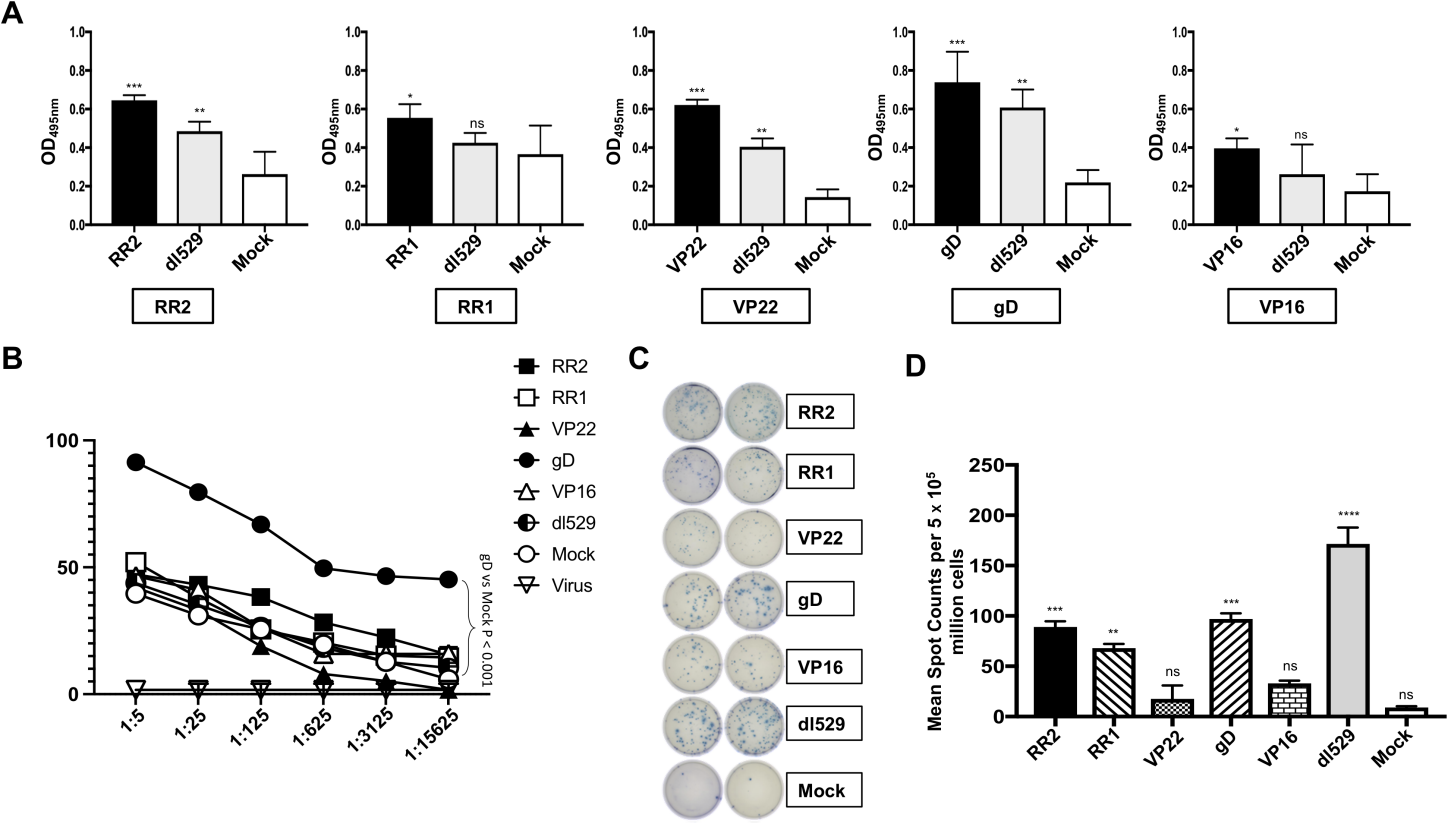
Formation of antibodies in HSV-2 infected guinea pigs following therapeutic immunization with five adenovirus-based vaccine candidates **(A)** Levels of antigen specific IgG detected in the guinea pigs vaccinated with Ad5 expressing RR2, RR1, gD, VP22 and VP16 antigens by ELISA. The sera were evaluated at 1:1000 dilution. **(B)** Neutralization assay among the vaccinated and mock-vaccinated groups showing vaccine-induced serum-neutralization. Comparison of the HSV-2 MS strain specific neutralizing antibodies induced in the guinea pigs vaccinated with Ad5 expressing RR2, RR1, gD, VP22 and VP16 antigens. **(C)**; left panel) Representative ELISpot images showing average frequencies of IFN-γ producing cell spots from mononuclear cells from VM tissue (1 x 10^5^ cells per well) of HSV-2 infected guinea pigs treated with different HSV-2 proteins namely RR2, RR1, VP22, gD, VP16. dl529 were used as a positive control. **(C)**; right panel) The bar diagrams show the average/mean numbers (+ SD) of IFN-γ-spot forming cells (SFCs) after stimulation with HSV-2 proteins in VM tissues of different groups of guinea pigs. A strong response is defined for mean SFCs > 25 per 1 x 10^5^ stimulated mononuclear cells. * = P<0.05, ** = P≤0.01, *** = P≤0.001, **** = P≤0.0001, ns, non significant.

Five weeks after the adenovirus immunization, immune sera were collected and tested for antigen-specific IgG by ELISA. Sera from Ad5-antigen vaccinated animals were evaluated at 1:1000 dilution and recognized their corresponding coating antigens. Significantly high levels of IgG specific to immunizing antigens were detected by ELISA in the serum of guinea pigs that were vaccinated with Ad5-antigen (**Figure 2A**). Immune sera from guinea pigs that were vaccinated with rAd-RR2, rAd-VP22, and rAd-gD had IgG that bound with high affinity to corresponding coated antigen in comparison to rAd-VP16, and rAd-RR1 (**Figure 2A**). Sera from dl529 vaccinated animals evaluated at 1:1000 dilution also recognized RR2, Vp22 and gD as coating antigens (**Figure 2A**). However, sera from mock-vaccinated animals displayed less titer (**Figure 2A**). Since the rAd-Ag induced high antibody titer than mock alone, we determined whether the Ad5-antigen would induce better HSV-2-specific neutralizing antibody titers. The serum samples were evaluated by neutralization assays against the HSV-2 MS strain. rAd-gD based vaccine elicited stronger serum neutralizing activity against the HSV-2 MS strain (**Figure 2B**). While serum from the other antigens rAd-RR2, rAd-RR1, rAd-VP22, and rAd-VP16 and mock vaccinated hamsters didn’t manifest strong neutralizing activity against HSV-2 MS strain (**Figure 2B**). These results suggest that the rAd-gD vaccine induced stronger neutralizing antibodies that prevented immune escape by the HSV-2. Furthermore, the highest frequencies ofIFN-γ-producing cells measured by ELISpot were detected in the VM cell suspensions of guinea pigs that were vaccinated with rAd-RR2, rAd-RR1, and rAd-gD while lesser spot forming cells were observed in VM cell suspension of guinea pigs that were immunized with rAd-VP22, and rAd-VP16 (**Figures 2C, D**). The mock-vaccinated animals displayed the lowest spot forming cells (**Figures 2C, D**).

### 2.3 Therapeutic vaccination of HSV-2 infected guinea pigs with rAd-RR2, rAd-RR1, and rAd-gD increased the frequencies of tissue-resident CD4^+^ and CD8^+^ T cells in the DRG and VM

Guinea pigs (n = 42) were infected intravaginally with 5 x 10^5^ pfu of HSV-2 (strain MS). Once the acute infection was resolved, latently infected animals were immunized with 10^10^ adenovirus antigen viral particles (VP) on day 15 post-infection. One of the groups was immunized with dl529 (positive control) while another group was treated with vector alone (adenovirus alone) and referred to as mock. On day 52 post-infection, guinea pigs were euthanized, single-cell suspensions from the VM tissue were obtained, and the DRG, VM and Spleen resident CD4^+^ and CD8^+^ T cells were analyzed. We observed a significantly higher frequencies of CD4^+^ cells in the rAd-RR2, rAd-RR1, rAd-gD, and rAd-VP16 vaccinated groups compared to those with the mock-vaccinated group (i.e., adjuvant alone) in the DRG tissues. (**Supplementary Figures S2A, B**: *top panel*). A significantly higher frequencies of CD8^+^ cells in the rAd-RR2, rAd-RR1, and rAd-gD vaccinated groups compared to those with the mock-vaccinated group (i.e., adjuvant alone) was observed in the DRG tissues. (**Supplementary Figures S2A, B**: *top panel*). rAd-VP22 did not show significantly higher frequency of CD4^+^ and CD8^+^ T cells compared to the mock-vaccinated group (**Supplementary Figures S2, B**: *top panel*). Also, rAd-VP16 had a lower frequency of CD8^+^ T cells compared to mock-vaccinated group (**Supplementary Figures S2A, B**: *top panel*). A significantly higher frequencies of CD4^+^ cells in the rAd-RR2 vaccinated groups compared to those with the mock-vaccinated group (i.e., adjuvant alone) was seen in the VM tissues (**Supplementary Figures S2A, B**: *middle panel*). rAd-RR1, rAd-gD, and rAd-VP16 showed relatively but not significant higher frequencies of CD4^+^ cells compared to mock (**Supplementary Figures S2A, B**: *middle panel*). We observed a significantly higher frequencies of CD8^+^ T cells in the rAd-RR2, rAd-VP22, and rAd-VP16) vaccinated groups compared to mock (**Supplementary Figures S2A, B**: *middle panel*). The spleen showed a significantly higher frequencies of CD4^+^ T cells in the rAd-RR2, rAd-RR1, rAd-VP22, rAd-gD, and rAd-VP16 vaccinated groups compared to mock (**Supplementary Figures S2A, B**: *lower panel*). A significantly higher frequencies of CD8^+^ T cells was observed in the rAd-RR2, rAd-VP22, and rAd-VP16 in vaccinated groups compared to mock (**Supplementary Figures S2A, B**: *lower panel*).

### 2.4 Therapeutic vaccination of HSV-2 infected guinea pigs with rAd-RR2, rAd-RR1 and rAd-gD boosted memory T cells in the DRG and VM

**Figure 3:**
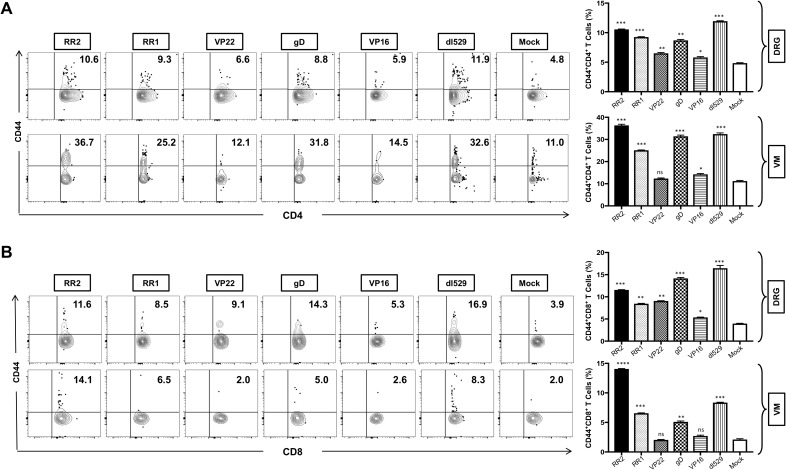
Increased frequencies of CD4^+^CD44^+^ T cells and CD8^+^CD44^+^ T cells in the DRG and VM of HSV-2 infected guinea pigs following therapeutic immunization with five adenovirus- based vaccine candidates: **(A)** Representative FACS data (left panel) and average frequencies (right panel) of CD4^+^CD44^+^ T cells detected in the DRG, VM of vaccinated and mock-vaccinated animals. **(B)** Representative FACS data (left panel) and average frequencies (right panel) of CD8^+^CD44^+^ T cells detected in the DRG, and VM of vaccinated and mock-vaccinated animals. * = P<0.05, ** = P≤0.01, *** = P≤0.001, **** = P≤0.0001, ns, non significant.

We next sought to determine the generation of total memory cells in the VM and DRG tissue of HSV-2-infected and vaccinated guinea pigs. The total memory cells in the VM and DRG was analyzed by observing the expression of CD44 memory cell marker on CD4^+^ T and CD8^+^ T cells by FACS. Significantly higher frequencies of CD4^+^CD44^+^ T cells were induced in the DRG by rAd-RR2, rAd-RR1, followed by rAd-gD, rAd-VP22 and rAd-VP16 vaccinated groups compared to mock (**Figure 3A**: *top panel*). However, higher frequencies of CD4^+^CD44^+^ T cells were induced in the VM of rAd-RR2, rAd-RR1 and rAd-gD followed by rAd-VP16 vaccinated groups compared to mock (**Figure 3A**: *bottom panel*). rAd-RR2, rAd-RR1, rAd-gD, rAd-VP22 and rAd-VP16 vaccinated groups showed higher frequencies of CD8^+^CD44^+^ T cells compared to mock in the DRG of guinea pigs (**Figure 3B**: *top panel*). However, only rAd-RR2, rAd-RR1, and rAd-gD showed higher frequencies of CD8^+^CD44^+^ T cells compared to mock in the VM of guinea pigs (**Figure 3A**: *bottom panel*). An increase in CD44^+^ T cells correlates with the efficacy of the vaccine in inducing long-term adaptive immunity.

### 2.5 Therapeutic vaccination of HSV-2 infected guinea pigs with rAd-antigen efficiently boosted tissue-resident memory in the DRG and VM

**Figure 4:**
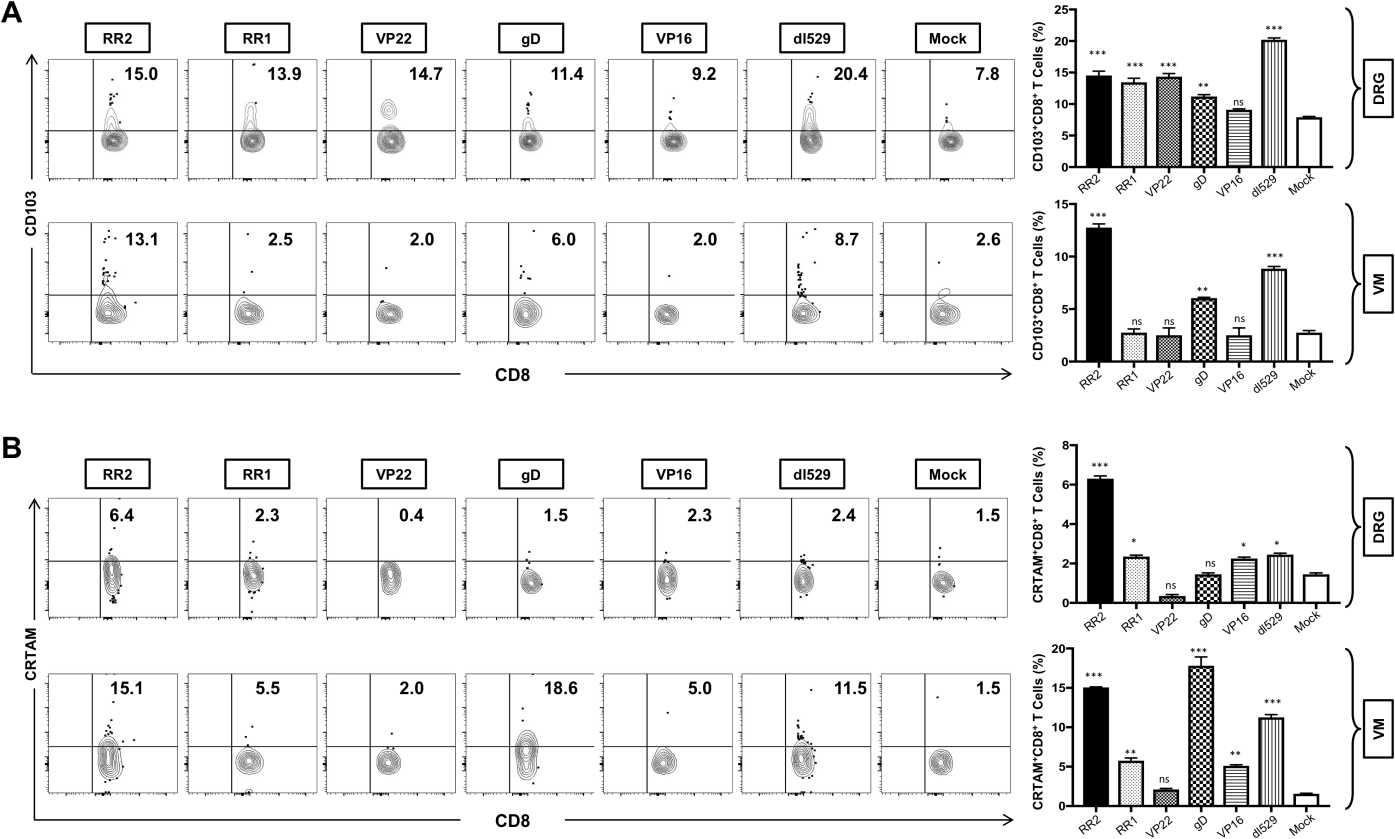
Increased frequencies of CD8^+^CD103^+^ T cells and CD8^+^CRTAM T cells in the DRG and VM of HSV-2 infected guinea pigs following therapeutic immunization with five adenovirus-based vaccine candidates: **(A)** Representative FACS data (left panel) and average frequencies (right panel) of CD8^+^CD103^+^ T cells detected in the DRG, and VM of vaccinated and mock-vaccinated animals. **(B)** Representative FACS data (left panel) and average frequencies (right panel) of CD8^+^CRTAM T cells detected in the DRG, and VM of vaccinated and mock-vaccinated animals. * = P<0.05, ** = P≤0.01, *** = P≤0.001, ns, non significant.

We next determined the association of various protection parameters (i.e., virus shedding and severity and frequency of recurrent genital herpes lesions) with tissue-resident memory that resides at the vaginal mucocutaneous and DRG tissue of HSV-2-infected and vaccinated guinea pigs. The resident T cells in the VM and DRG were analyzed by observing the expression of CD103 on CD8^+^T by FACS. Significantly higher frequencies of CD8^+^CD103^+^ T cells were induced in the DRG by vaccination of rAd-RR2, rAd-RR1, rAd-gD, and rAd-VP22 compared to mock (**Figure 4A**: *top panel*). However, higher frequencies of CD8^+^CD103^+^ T cells were induced only in the VM of rAd-RR2 and rAd-gD vaccinated groups compared to mock (**Figure 4A**: *bottom panel*). CD103 is a marker for tissue-resident memory T-cells and binds to E-cadherin to facilitate the retention of T cells within epithelial cells. This allows the resident immune cells to provide rapid and localized immune response upon virus entry or reactivation. The higher frequencies of CD8^+^CD103^+^ T cells in theDRG and VM of rAd-RR2 and rAd-gD vaccinated groups correlates with the efficacy of the rAd-Ag expressing gD and RR2 protein.

### 2.6 Therapeutic vaccination of HSV-2 infected guinea pigs with rAd-antigen \activated T cells in the DRG and VM

CD69 and CRTAM are rapidly expressed on the cell surface following activation and are used to identify activated immune cells. CRTAM expression on activated CD8^+^ T cells promotes the production of cytotoxic granules (e.g., granzyme B, perforin) and cytokines like IFN-γ, enhancing their ability to kill infected cells. We observed a significantly higher frequencies of CD8^+^CRTAM T cells in the DRG of guinea pigs vaccinated with rAd-RR2, rAd-RR1, and rAd-VP16 compared to mock (**Figure 4B**: *top panel*). Also, higher frequencies of CD8^+^CRTAM T cells were induced in the VM of rAd-RR2, rAd-RR1 and rAd-gD followed by rAd-VP16 vaccinated groups compared to mock (**Figure 4B**: *bottom panel*).

Further, the activation status in the VM and DRG was analyzed by observing the expression of CD69 marker on CD4^+^ T and CD8^+^ T cells by FACS. Significantly higher frequencies of CD4^+^CD69^+^ T cells were induced in the DRG by rAd-RR2, rAd-RR1, rAd-gD, rAd-VP22 and rAd-VP16 vaccinated groups compared to mock (**Supplementary Figure S3A**: *top panel*). However, higher frequencies of CD4^+^CD69^+^ T cells were induced in the VM of rAd-RR2, rAd-RR1 and rAd-gD followed by rAd-VP16 vaccinated groups compared to mock (**Supplementary Figure S3A**: *bottom panel*). rAd-RR2, rAd-RR1, rAd-gD, and rAd-VP22 vaccinated groups showed higher frequencies of CD8^+^CD69^+^ T cells compared to mock in the DRG of guinea pigs (**Supplementary Figure S3B**: *top panel*). However, only rAd-RR2, and rAd-gD followed by rAd-RR1, and rAd-VP22 showed higher frequencies of CD8^+^CD69^+^ T cells compared to mock in the VM of guinea pigs (**Supplementary Figure S3B**: *bottom panel*).

### 2.7 Induced protection from HSV-2 infection following therapeutic rAd-antigen vaccination is associated with more functional tissue-resident IFN-γ+</sup>TNF-α+</sup>Ki67 CD4 and CD8^+^ T cells

We next compared the function of CD4^+^ and CD8^+^ T cells in the DRG, and VM, of HSV-2-infected guinea pigs following rAd-antigen vaccination. On day 52, after the immunization guinea pigs were euthanized, and single-cell suspensions from the DRG and VM tissues were obtained, and the function of DRG-resident and VM-resident T cells was analyzed by production of Ki67, IFN-γ and TNF-α expression by FACS.

**Figure 5:**
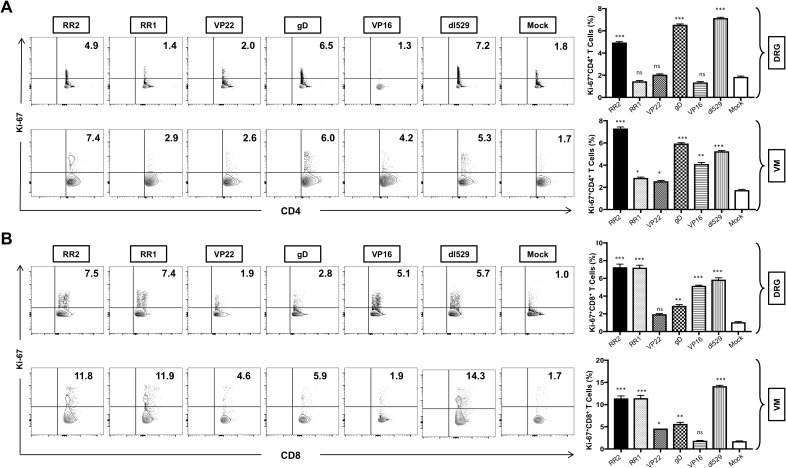
Increased frequencies of CD4^+^Ki-67 T cells and CD8^+^Ki-67 T cells in the DRG and VM of HSV-2 infected guinea pigs following therapeutic immunization with five adenovirus-based vaccine candidates: **(A)** Representative FACS data (left panel) and average frequencies (right panel) of CD4^+^Ki-67 T cells detected in the DRG, and VM of vaccinated and mock-vaccinated animals. **(B)** Representative FACS data (left panel) and average frequencies (right panel) of CD8^+^Ki-67 T cells detected in the DRG, and VM of vaccinated and mock-vaccinated animals. * = P<0.05, ** = P≤0.01, *** = P≤0.001, ns, non significant.

Significantly higher frequencies of CD4^+^Ki67^+^ T cells were induced in the DRG of rAd-RR2 and rAd-gD vaccinated groups compared to mock (**Figure 5A**: *top panel*). However, higher frequencies of CD4^+^Ki67^+^ T cells were induced in the VM of rAd-RR2 and rAd-gD followed by rAd-VP16, rAd-RR1 and rAd-VP22 vaccinated groups compared to mock (**Figure 5A**: *bottom panel*). Similarly, rAd-RR2, rAd-RR1, rAd-gD, and rAd-VP16 vaccinated groups showed higher frequencies of CD8^+^ Ki67^+^ T cells compared to mock in the DRG of guinea pigs (**Figure 5B**: *top panel*). However, rAd-RR2, rAd-RR1, rAd-gD, and rAd-VP22 showed higher frequencies of CD8^+^ Ki67^+^ T cells compared to mock in the VM of guinea pigs (**Figure 5B**: *bottom panel*). Conversely, rAd-VP16 did not show any higher frequencies of CD8^+^ Ki67^+^ T cells compared to mock in the VM of guinea pigs (**Figure 5B**: *bottom panel*).

**Figure 6:**
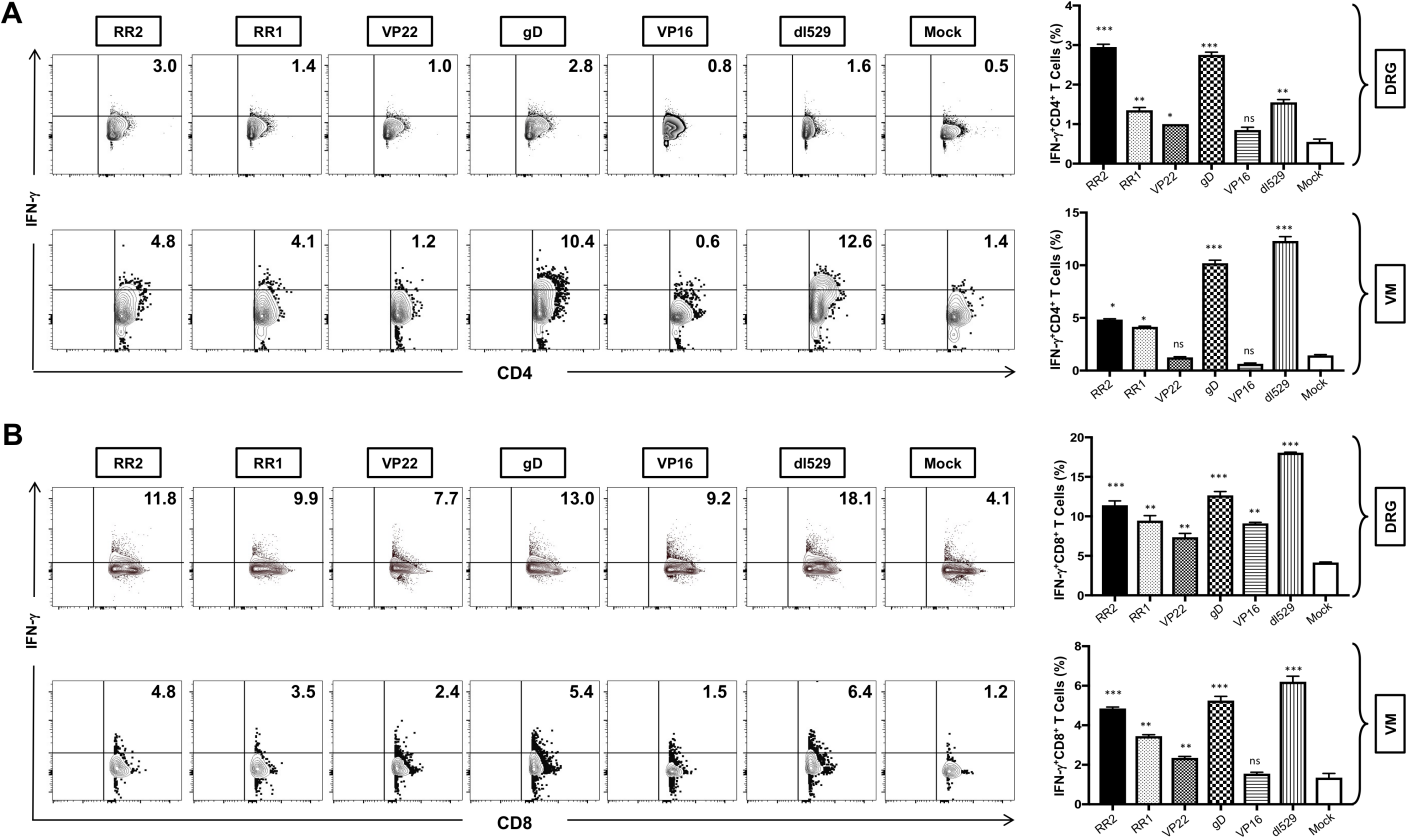
Increased frequencies of CD4^+^IFN-γ T cells and CD8^+^ IFN-γ T cells in the DRG and VM of HSV-2 infected guinea pigs following therapeutic immunization with five adenovirus-based vaccine candidates: **(A)** Representative FACS data (left panel) and average frequencies (right panel) of CD4^+^ IFN-γ T cells detected in the DRG, and VM of vaccinated and mock-vaccinated animals. **(B)** Representative FACS data (left panel) and average frequencies (right panel) of CD8^+^ IFN-γ T cells detected in the DRG, and VM of vaccinated and mock-vaccinated animals.

rAd-RR2, rAd-RR1, rAd-gD, and rAd-VP22 vaccinated groups displayed high frequencies of CD4^+^IFN-γ^+^T cells in the DRG (**Figure 6A**: *top panel*). While rAd-VP16, and rAd-VP22 vaccinated groups displayed low frequencies of CD4^+^IFN-γ^+^T cells in the VM (**Figure 6A**: *bottom panel*). However, all rAD-antigen groups displayed high frequencies of CD8^+^IFN-γ^+^T cells in the DRG (**Figure 6B**: *top panel*). While rAd-VP16, showed low frequencies of CD8^+^IFN-γ^+^T cells in the VM (**Figure 6B**: *bottom panel*). Similarly, CD4^+^TNF-α was exhibited by all rAD-antigen groups in the DRG (**Supplementary Figure S4A**: *top panel*).). While only rAd-RR2, and rAd-gD displayed higher frequencies of CD4^+^ TNF-α in the VM ((**Supplementary Figure S4A**: *bottom panel*). Likewise, all rAD-antigen groups except rAd-VP16 displayed high frequency of CD8^+^TNF-α T cells in the DRG and VM (**Supplementary Figure S4B**). An increase in Ki-67^+^ T cells not only corelates with cell activation and expansion but also persistence, functionality, and memory formation. correlating with effector function and pathogen clearance. While IFN-γ^+^TNF-α^+^T cells provide effector function and pathogen clearance and an increase in these markers is indicative of an active immune response following vaccination. Thus, establishing rAD-Ag especially rAd-RR2, and rAd-gD as adenoviral vaccines for controlling viral infection. Further, statistical comparisons of RR2 antigen vs. all other immunizations groups in this study has been provided in **Supplementary Table 1**.

## 3 Discussion

HSV-2 infection affects more than 500 million people worldwide and increases the risk of HIV acquisition and transmission. The health and socioeconomic burden concerning genital herpes highlights the need for a therapeutic herpes vaccine that can alleviate the disease impact (25, 26). Therapeutic vaccines targeted towards HSV-2 infected people will reduce both symptomatic and asymptomatic genital HSV-2 shedding and reduce the risk of transmission of both HSV and HSV-associated viral diseases like HIV (27). Extensive research in both animal models (mouse, guinea pig) and human studies have led to an improved but incomplete understanding of the immune responses that need to be stimulated by a vaccine. These include HSV antigen-specific neutralizing antibody responses, which are thought to be particularly important for a prophylactic vaccine (27). Adaptive cellular immune responses have been shown to be important for clearance of the virus in people with HSV infection, and stimulation of these responses are essential for therapeutic vaccines (28, 29). In addition, stimulation of mucosal immune responses at the site of infection is important for both prophylactic and therapeutic vaccines (27).

Vaccine strategies for therapeutic as well as prophylactic vaccination include subunit vaccines, attenuated or replication deficient virus, and DNA-based vaccines Therapeutic herpes vaccines aim to reduce recurrences and viral shedding but have shown mixed clinical outcomes (30). Overall, viral latency and immune evasion continue to hinder HSV vaccine development (7). Live-attenuated vaccines (e.g., ΔgD-2) elicit strong immune responses but pose safety risks, especially in immunocompromised individuals (25). Inactivated vaccines have been largely abandoned due to poor efficacy (31). Subunit vaccines, like GSK’s gD2 (Herpevac) and GEN-003, offer safety but have shown limited clinical success, particularly in preventing HSV-2 infection (32). DNA vaccines, on the other hand, are safe and capable of inducing immune responses but face challenges with human immunogenicity (33). Viral vector-based vaccines, including adenovirus-based platforms, show promise with robust immunity (33). Following recent mRNA-based vaccine success against COVID-19, mRNA-based genital herpes vaccines showed success in pre-clinical animal vaccine studies (33, 34) and are being currently (2023-2025) explored in a couple of clinical trials (by BioNTech/Moderna and Pfizer), though the results of these clinical trials remain to be seen. In our previous study, we showed that immunizing guinea pigs with an immunogenic HSV-2 protein RR2 and treatment with adenovirus-expressing chemokine provide better protection against recurrent genital herpes. This strategy helped to prevent the migration of HSV-2 from mucosa to neurons leading to decreased reactivation and viral shedding (15, 16). Subunit vaccines target HSV surface glycoproteins and generate strong neutralizing antibody responses (34). These glycoproteins are commonly targeted by the immune system; however, they did not provide sufficient protection against recurrent herpetic disease when included in the vaccine (35, 36). HSV-2-based glycoprotein-subunit vaccines failed to provide adequate antiviral protection in large-scale clinical trials as a result of the virus developing strategies to evade immune responses (37). Although these strategies have not produced a licensed vaccine, they have provided important information about correlates of protection and proof of concept to direct future HSV vaccine studies. Given that glycoprotein subunit vaccines have failed to prevent or reduce the symptoms of HSV-2 genital herpes in trials, perhaps it is time to consider the logical alternative of an adenovirus-based vaccine.

Many recently approved vaccines developed for SARS-CoV2 were based on immunization by spike-protein-encoding adenovirus vectors (AVs). AVs are a relatively new technology, although adenoviruses have been used as gene delivery vehicles since the earliest days of gene therapy. Adenoviruses are an important therapeutic vector due their well-defined biology, genetic stability, better transduction efficiency, and ease of mass production. Unlike lentiviruses, AVs remain episomal and do not incorporate into the host genome (38, 39). AVs are characterized by their high immunogenicity via both innate and adaptive inflammatory responses against the adenoviral capsid structures (39–41). About 50 serotypes of adenovirus have been isolated in humans, which are further sub-grouped into seven subtypes (A–G) depending on their homology sequence (42, 43). AVs’ wide tissue tropism, expression of the target antigen and their ability to trigger potent immunogenicity have been used to create vaccine candidates for cancer immunotherapies as well as infectious diseases such as Ebola disease, AIDS, Zika virus, tuberculosis, and malaria. Furthermore, the flexible viral biology allows us to engineer them to produce vaccines with increased efficacy (44). CanSino Biologics developed Convidecia (AD5-nCOV) using an Adenovirus type 5 (Ad5) vector with genome of SARS-CoV-2 Spike protein. A phase I dose escalation trial was carried out in healthy volunteers, 108 of whom were in the 18–60 age group, and results showed a safe and good tolerability profile (45). Ad-5 vectors represent a vaccine platform that has been tested for use against various infectious diseases, largely due to their safety and ability to stimulate robust cellular and/or humoral immune responses in both animal models and humans. Ad-5 have demonstrated efficient transduction, infect both dividing and nondividing cells, and their DNA is not incorporated into host cells’ chromosome, minimizing concerns about insertional mutagenesis.

Past clinical trials have suggested that a therapeutic herpes vaccine may be able to change the course of recurrent genital herpes disease (46). Our goal for a therapeutic HSV-2 vaccine was to reduce or prevent symptomatic recurrences as well as to stop or reduce asymptomatic viral shedding and transmission that would provide a public health benefit (15, 16). In the present study, we demonstrate that when given therapeutically in latently infected guinea pigs, the rAd-RR2, rAd-RR1, and rAd-gD-based vaccine significantly reduced the frequency of recurrent genital herpes over two months compared to the mock-vaccine. Virus-specific neutralizing antibody titers were significantly boosted in rAd-gD-based vaccinated protected animals compared to non-protected mock-vaccinated animals. However, as expected, no correlation was found between protection and virus-specific neutralizing antibody titers among the animals that were vaccinated with T-cell antigen-based vaccines rAd-RR2, rAd-RR1, suggesting that both effector T cells and functional neutralizing antibodies may be required for therapeutic protection against recurrent genital herpes.

An increased number and function of DRG-resident CD4^+^ and CD8^+^ T cells are associated with a reduction of HSV virus in the DRG, reduced severity, and rate of recurrent genital herpes in latently infected vaccinated guinea pigs. The presence of T cells near infected neurons affects the viral replication and/or reduced recurrences of HSV-2 from latency, which in turn reduces the severity of recurrent genital herpes (47). Moreover, the presence and increased number of functional CD4^+^ and CD8^+^ T cells in vaginal mucosa also affects viral replication at the entry site (48). To address this, we evaluated the therapeutic efficacy of recombinant adenoviruses in a guinea pig model, which is capable of expressing full length envelope glycoprotein D (gD), the tegument protein VP22 (encoded by the UL49 gene) and VP16 (encoded by the UL48 gene), and ribonucleotide reductase subunit 1& 2 protein (RR1; encoded by the UL39 gene & RR2; encoded by the UL40 gene). Recombinant adenovirus-based vaccines exhibited protection against HSV infection and generated strong antigen-specific T-cell responses. Moreover, the severity of genital lesions in guinea pigs immunized with rAd antigens was significantly decreased. Compared with rAd-gd and rAd-RR2; rAd-VP22 and rAd-VP16 showed lesser protection against the intravaginal HSV-2 challenge.

Several studies have shown that proinflammatory cytokines like IFN-γ and TNF-α are important for the resolution of lesions and clearance of HSV-2 genital infections (49). Recombinant adenovirus vaccines have ability to induce cellular immunity which is important in controlling viral reactivation and HSV disease. We used the replication-defective adenovirus serotype 5 (rAd5) vector as: (i) it has remarkable ability to boost mucosal CD8^+^ T cell responses (ii) its ability to elicit a considerably potent and long-lasting pathogen-specific CD8^+^ T cell response in humans (iii) its natural tropism for mucosal tissues (50).

In the present study, HSV proteins that were highly recognized by the immune system from naturally protected asymptomatic individuals were selected as therapeutic vaccine candidates (16). The safety, immunogenicity, and protective efficacy of these HSV-2 proteins have been assessed in a preclinical therapeutic vaccine trial using the guinea pig model of recurrent genital herpes (16). Comparable to the dl5-29, the therapeutic immunization with rAd-gD, rAd-RR1, rAd-RR2, rAd—VP16 and rAd-VP22, produced significant protection against recurrent genital herpes infection and disease. RR2 has been previously reported as a major target of HSV-2 specific CD8^+^ T cells in humans (16, 51). The HSV-2 ribonucleotide reductase is a major target of HSV-2-specific CD8^+^ T cells in humans. It consists of two heterologous protein subunits. The small subunit (RR2) is a 38-kDa protein encoded by the UL40 gene, and the large subunit (RR1), designated ICP10, is a 140-kDa protein encoded by the UL39 gene (15). RR2 protein can boost neutralizing antibodies and increase the numbers of functional IFN-γ^+^CRTAM^+^ CD8^+^ T cells within the VM tissues (15, 16). HSV-2’s glycoproteins have been the focus of vaccine development efforts for 30 years, gD being a dominant antigen. Glycoprotein D is a common target for most vaccines as it is essential for viral entry into host cells. The glycoprotein D2 vaccine by GlaxoSmithKline is a subunit vaccine consisting of the HSV-2 glycoprotein D (gD2-AS04) with adjuvants (52). GEN-003 is a subunit vaccine with a transmembrane deletion mutant of glycoprotein D. It was shown to stimulate both humoral and cellular responses, with neutralizing antibodies and T-cell responses (53). G103 is formulated with recombinant HSV-2 protein gD with TLR4 agonist glucopyranosyl lipid A adjuvant (54).

The present study demonstrates that intravaginal vaccination of guinea pigs with rAD-antigen increased the number of functional tissue-resident IFN-γ-producing CD69^+^ CD44^+^ CD4^+^ and CRTAM^+^ CD103^+^ CD8^+^ TRM cells in the vaginal mucosa and protected against spontaneous recurrent genital herpes in the infected guinea pigs. These robust local B- and T-cell responses were associated with a significant reduction in both virus shedding and the severity and frequency of recurrent genital herpes lesions. Interestingly, therapeutic immunization with rAdVP22 and rAd-VP16 did not result in a more robust protection than with rAd-RR2, rAd-RR1 and rAd-gD. These preclinical findings demonstrate that rAd-RR2, rAd-RR1 and rAd-gD are viable vaccine candidates to be incorporated in the next genital herpes therapeutic mucosal vaccine to be clinically tested. Nevertheless, it is interesting that the protective efficacy of the rAd-RR2 and rAd-gD vaccine was comparable to that induced with the dl5-29. The rAd-RR2 and rAd-gD demonstrate a viable B- and T-cell candidate antigen to be incorporated in future genital herpes therapeutic mucosal vaccines.

In conclusion, our study demonstrates that immunizing guinea pigs with an immunogenic rAd-RR2 and rAd-gD vaccine provides better protection against recurrent genital herpes. The use of recombinant adenovirus seems to produce the required immune response that leads to decreased reactivation and viral shedding. The current literature suggests that CD8+ T cells reduce neuronal infection or viral replication within neurons (55). Recombinant adenoviruses elicit a robust cellular and humoral immunity against the encoded antigen and vaccination with rAd can produce effective anti-viral immunity (56). To date, no study has reported the role of rAd-RR2 and rAd-gD vaccine in attracting more functional DRG-resident CD4+ and CD8+ T cells which in turn affects recurrent genital herpes infection and disease. In the present study, we showed that increased number and function of DRG-resident CD4+ and CD8+ T cells were associated with a reduction in the HSV-2 DNA copy numbers in the DRG, reduced severity, and rate of recurrent genital herpes in latently infected vaccinated guinea pigs. The presence of T cells near infected neurons may likely affect the viral replication and/or reduced recurrences of HSV-2 from latency, which in turn reduces the severity of recurrent genital herpes. Moreover, the presence and increased number of functional CD4+ and CD8+ T cells in vaginal mucosa may also affect viral replication at the entry site. However, providing mechanistic evidence of the direct implication of DRG- and VM-resident CD4+ and CD8+ T cells in reduction of spontaneous active replication would require extensive *in vivo* and *in vitro* studies, and this will be the subject of future reports.

## 4 Materials & methods

### 4.1 Animals

Female guinea pigs (Hartley strain, Charles River Laboratories, San Diego, CA) weighing 275-350 g (5-6 weeks old) were housed at the University of California, Irvine vivarium. The Institutional Animal Care and Use Committee of the University of California, Irvine, reviewed and approved the protocol for these studies (IACUC # AUP-22-086).

### 4.2 Virus generation

Adenovirus type 5 (rAd5) vaccines containing the full-length genes of HSV-2 virus were generated based on the published antigen sequences of HSV-2 genes and expressed under control of the cytomegalovirus promoter synthesized by Vector Builder Inc (Chicago, IL, USA). The vaccine constructs expressed the published DNA sequence of HSV-2 publicly available as GenBank accession no. MK855052.1. These sequences were inserted into a recombinant plasmid containing rAd5 sequence that is deleted in E1 and E3 genes. The respective transgenes were cloned into the E1 region that additionally contains a downstream molecular dsRNA adjuvant in the gene cassette and is expressed together with the transgene in the target cell. rAd5 particles were generated by transfection of transgene containing DNA into Expi293F cells (ThermoFisher Scientific) to generate rAd5 virions which were purified by CsCl density centrifugation.

### 4.3 Infection and immunization of guinea pigs

Throughout this study, we used the MS strain of HSV-2, generously gifted by Dr. David Bernstein (Cincinnati Children’s Hospital Medical Center, University of Cincinnati, OH). Guinea pigs (n = 42) were infected intravaginally with 5 x 10^5^ pfu of HSV-2 (strain MS). The virus was diluted to a final volume of 100μl using dPBS and administered using a pipette. The viral delivery was made possible using a butterfly infusion tubing (BD Vacutainer-368656) attached to the pipette tip. Once the acute infection was resolved, latently infected animals were separated into 7 groups (6 animals each) and vaccinated with 10^10^ adenoviral copies intravaginally in the right hind calf muscle on day 15 post-infection. BD 1 ml Tuberculin (TB) syringe 25G was used for intravaginal injection of Adenovirus type 5 expressing RR2, RR1, gD, VP22 and VP16 proteins (rAd-RR2, rAd-RR1, rAd-gD, rAd-VP22, and rAd-VP16). The adenovirus was diluted to a final 100 μl/guinea pig volume using dPBS. Dl529 treated animals were used as positive control while the mock group was treated with adenovirus vector alone.

### 4.4 Monitoring of recurrent HSV-2 disease in guinea pigs

To study genital HSV-2 infection, female guinea pigs were infected intravaginally with HSV-2. The acute (primary) genital lesions resolve by two weeks, followed by spontaneous reoccurrence over the ensuing months (recurrent phase). Viral shedding may be evidenced without even visible lesions by qPCR. Guinea pigs were examined for vaginal lesions, which were recorded for each animal every alternate day starting from day 22 until day 52 post-challenge.

### 4.5 Real-time qPCR for HSV-2 quantification from vaginal swabs and dorsal root ganglia

Vaginal swabs were collected every alternate day using a Dacron swab (type 1; Spectrum Laboratories, Los Angeles, CA) starting from day 25 until day 52 post-challenge. Individual swabs were transferred to a 2 mL sterile cryogenic vial containing 500ul culture medium and stored at -80oC until use. On day 52 post-challenge, VM and twelve lower lumbar and sacral dorsal root ganglia (DRG) per guinea pig were collected by cutting through the lumbar end of the spine. DNA was isolated from the collected vaginal swab, VM and DRG of guinea pigs by using DNeasy blood and tissue kits (Qiagen) (57). The presence of HSV-2 DNA was quantified by real-time PCR with 100 ng DNA (21). HSV-2 DNA copy number was determined using purified HSV-2 DNA (Advanced Biotechnologies, Columbia, MD). Primer sequences for HSV-2 Us9 were primer forward, 5′-GGCAGAAGCCTACTACTCGGAAA-3′, and reverse 5′-CCATGCGCACGAGGAAGT-3′ (32, 48, 49).

### 4.6 Splenocyte isolation

Spleens were harvested from guinea pigs at 52 days post-infection. Spleens were placed in 10 ml of cold PBS with 10% fetal bovine serum (FBS) and 2X antibiotic–antimycotic (Life Technologies, Carlsbad, CA). Spleens were minced finely and sequentially passed through a 100 µm mesh and a 70 µm mesh (BD Biosciences, San Jose, CA). Cells were then pelleted via centrifugation at 400 × g for 10 minutes at 4°C. Red blood cells were lysed using a lysis buffer and washed again. Isolated splenocytes were diluted to 1 × 10^6^ viable cells per ml in RPMI media with 10% (v/v) FBS and 2 × antibiotic–antimycotic. Viability was determined by Trypan blue staining.

### 4.7 Isolation of lymphocytes from the guinea pig’s vaginal mucosa and dorsal root ganglia

Vaginal mucosa and DRG were minced into fine pieces on a Petri Dish using a fine scalpel. The tissue was subjected to collagenase (7mg/ml) treatment and allowed to digest at 37°C for one hour on a rocker. After one hour, the digested tissue suspension was passed through a 100 μm cell strainer, followed by centrifugation and washing with RPMI media containing 10% FBS. Lymphocytes in the cell pellets were then suspended in 40% percoll, layered on top of 70% percoll, and centrifuged at 900 x g at room temperature for 30 minutes with the brake-off. The lymphocytes at the interface layer between 40% and 70% Percoll layers were harvested, washed with three volumes of RPMI, and spun down at 740 x g.

### 4.8 Enzyme-linked immunosorbent assay (ELISA and neutralizing antibodies

Blood (5 ml) was drawn from each guinea pig into yellow-top Vacutainer tubes (Becton, Dickinson). Sera were isolated by centrifugation for 10 min at 800 × g. For measuring antigen-specific antibody titers in the sera of immunized guinea pigs, ELISA plates were coated with 50 ng of individual HSV-2 proteins and incubated with guinea pig serum at a 1:1000 dilution, followed by horseradish peroxidase (HRP)-conjugated anti-guinea pig IgG (15, 36). Neutralizing antibody titers were determined by incubating 400 PFU of HSV-2 strain MS with serial dilutions of serum starting at 1:5 for 30 minutes at 37°C.

### 4.9 Flow cytometry analysis

Vaginal mucosa cells and splenocytes were analyzed by flow cytometry using the following antibodies: mouse anti-guinea pig CD8 (clone MCA752F, Bio-Rad Laboratories, Hercules, CA), mouse anti-guinea pig CD4 (clone MCA749PE, Bio-Rad Laboratories), anti-mouse CRTAM (clone 11-5, Biolegend, San Diego, CA), anti-mouse/human CD44 (clone IM7, Biolegend), anti-mouse CD69 (clone H1.2F3, BD Biosciences, San Jose, CA), and anti-mouse CD103 (clone 2E7, Biolegend) (13). For surface staining, mAbs against various cell markers were added to a total of 1X106 cells in phosphate-buffered saline containing 1% FBS and 0.1% sodium azide (fluorescence-activated cell sorter [FACS] buffer) and left for 45 minutes at 4°C. At the end of the incubation period, the cells were washed twice with FACS buffer. A total of 100,000 events were acquired by the LSRII (Becton Dickinson, Mountain View, CA), followed by analysis using FlowJo version 10 software (TreeStar, Ashland, OR).

### 4.10 Statistical analyses

Data for each assay were compared by analysis of variance (ANOVA) using GraphPad Prism version 10.1.0. Differences between the groups were identified by ANOVA and multiple comparison procedures. Data are expressed as the mean + SD. Results were considered statistically significant at P < 0.05.

## Data Availability

The datasets presented in this study can be found in online repositories. The names of the repository/repositories and accession number(s) can be found in the article/**Supplementary Material.**

## Supplementary material

Supplementary Figure 1 Schematic representation of the Ad5 vectors expressing RR1, RR2, VP16, VP22, and gD proteins of HSV-2.

Supplementary Figure 2 Frequency of CD4 and CD8 T cells in the vaginal mucosa of HSV-2 infected guinea pigs following therapeutic immunization with five adenovirus-based vaccine candidates **(A)** Fifty-six days post-infection, guinea pigs were euthanized, and single-cell suspensions from the DRG, vaginal mucosa (VM), and spleen were obtained after collagenase treatment. The DRG, VM, and spleen cells were stained for CD8^+^ and CD4^+^ expressing T cells and then analyzed by FACS. Representative FACS data **(A)** and average frequencies **(B)** of CD8^+^ CD4^+^ T cells detected in the DRG (top panel), VM (middle panel) and spleen (lower panel) of vaccinated animals, dl529 vaccinated and mock-vaccinated animals. Cells were analyzed using a BD LSR Fortessa Flow Cytometry system with 1 x 10^6^ events. The indicated P values performed by one-way ANOVA for significance show statistical significance between vaccinated versus mock-vaccinated control groups.

Supplementary Figure 3 Increased frequencies of CD4^+^CD69^+^ T cells and CD8^+^CD69^+^ T cells in the DRG and VM of HSV-2 infected guinea pigs following therapeutic immunization with five adenovirus- based vaccine candidates: **(A)** Representative FACS data (left panel) and average frequencies (right panel) of CD4^+^CD69^+^ T cells detected in the DRG, and VM of vaccinated and mock-vaccinated animals. **(B)** Representative FACS data (left panel) and average frequencies (right panel) of CD8^+^CD69^+^ T cells detected in the DRG, and VM of vaccinated and mock-vaccinated animals.

Supplementary Figure 4 Increased frequencies of CD4^+^IFN-γ T cells and CD8^+^ IFN-γ T cells in the DRG and VM of HSV-2 infected guinea pigs following therapeutic immunization with five adenovirus-based vaccine candidates: **(A)** Representative FACS data (left panel) and average frequencies (right panel) of CD4^+^ TNF-α T cells detected in the DRG, and VM of vaccinated and mock-vaccinated animals. (B) Representative FACS data (left panel) and average frequencies (right panel) of CD8^+^ TNF-α T cells detected in the DRG, and VM of vaccinated and mock-vaccinated animals.
