# Supplementary figures and images for: Therapeutic mucosal vaccination of herpes simplex virus type 2 infected guinea pigs with an adenovirus-based vaccine expressing the ribonucleotide reductase 2 and glycoprotein D induces local tissue-resident CD4+ and CD8+ TRM cells associated with protection against recurrent genital herpes
Source: Front Immunol. 2025 Mar 26;16:1568258. doi: 10.3389/fimmu.2025.1568258 (PMC11979635; doi:10.3389/fimmu.2025.1568258)

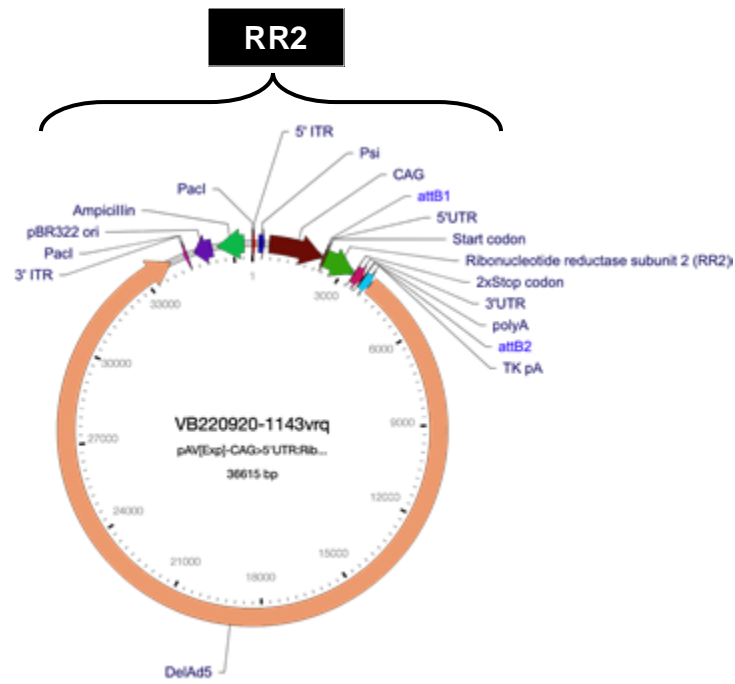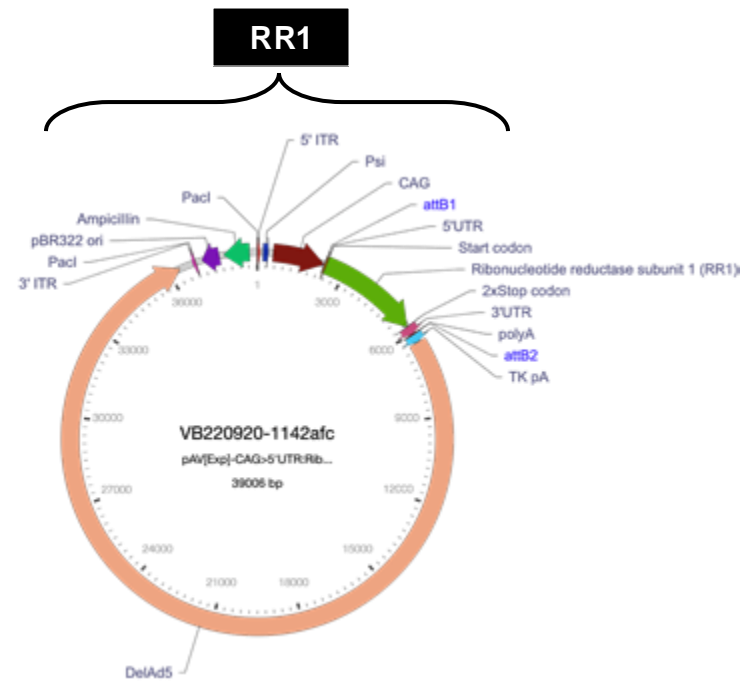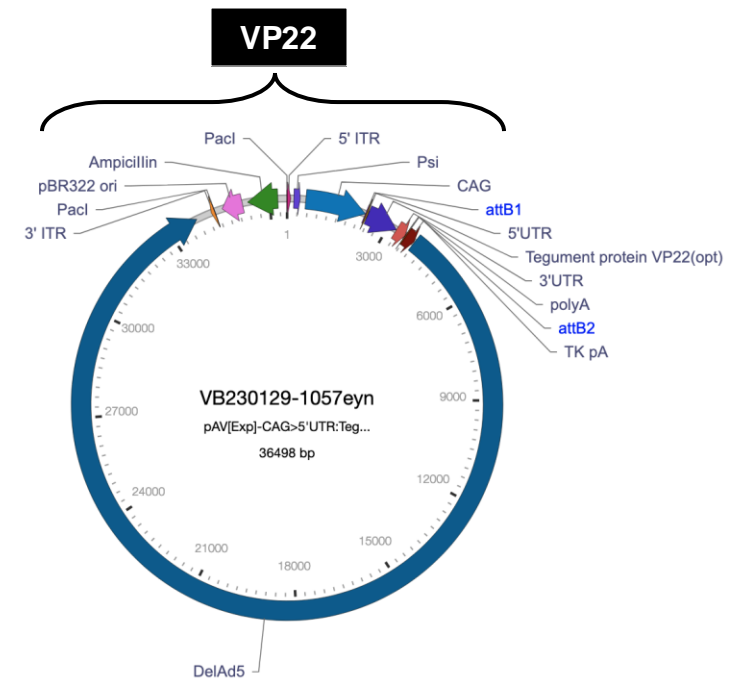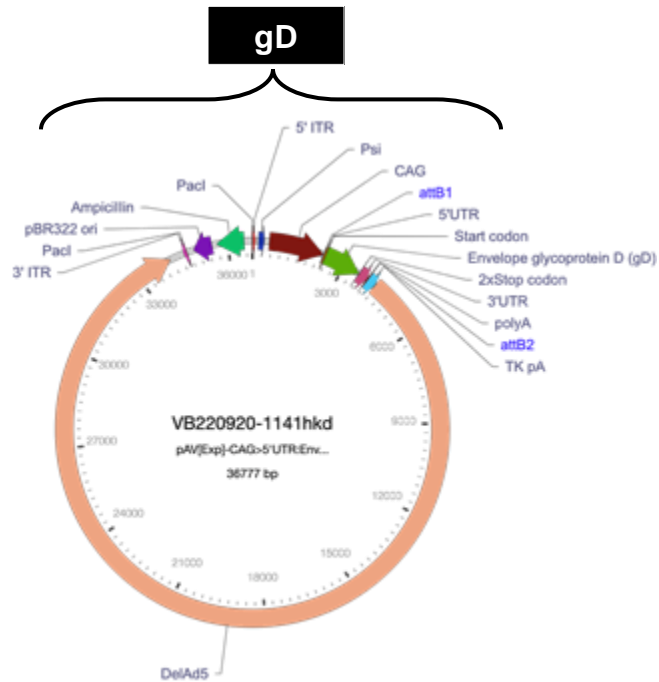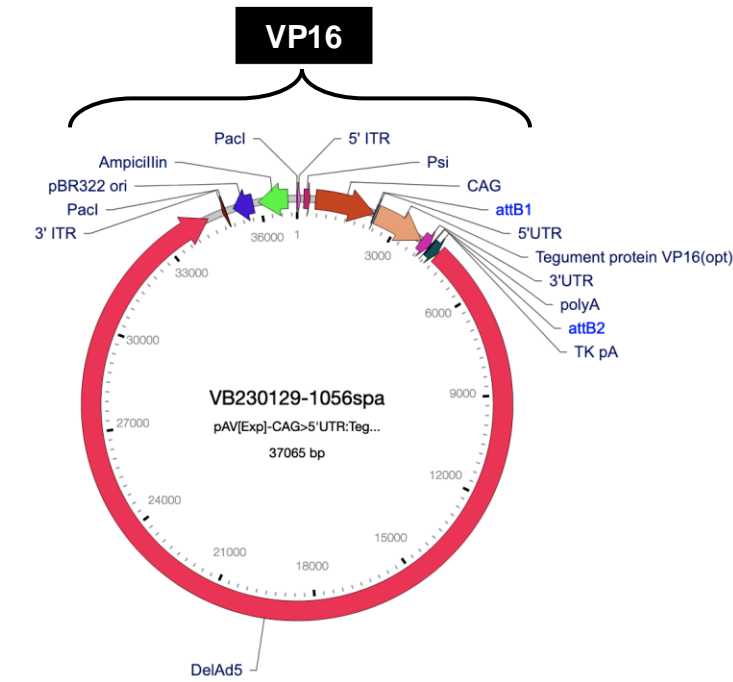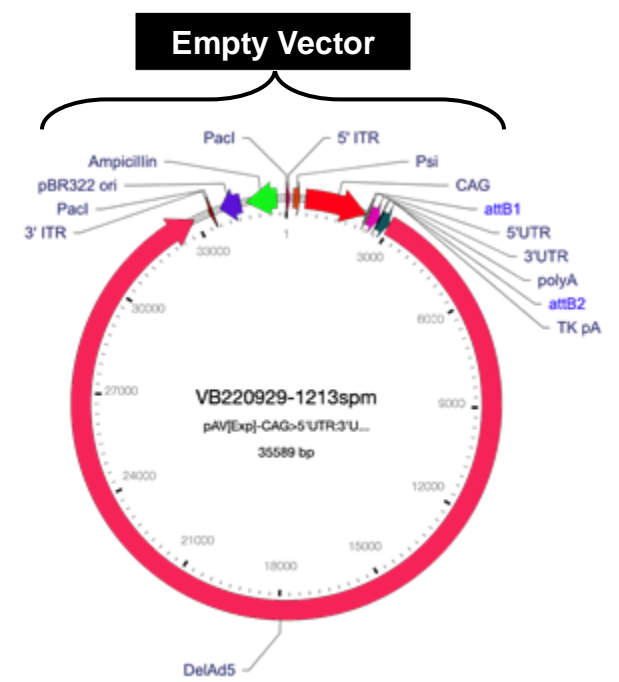

**A**

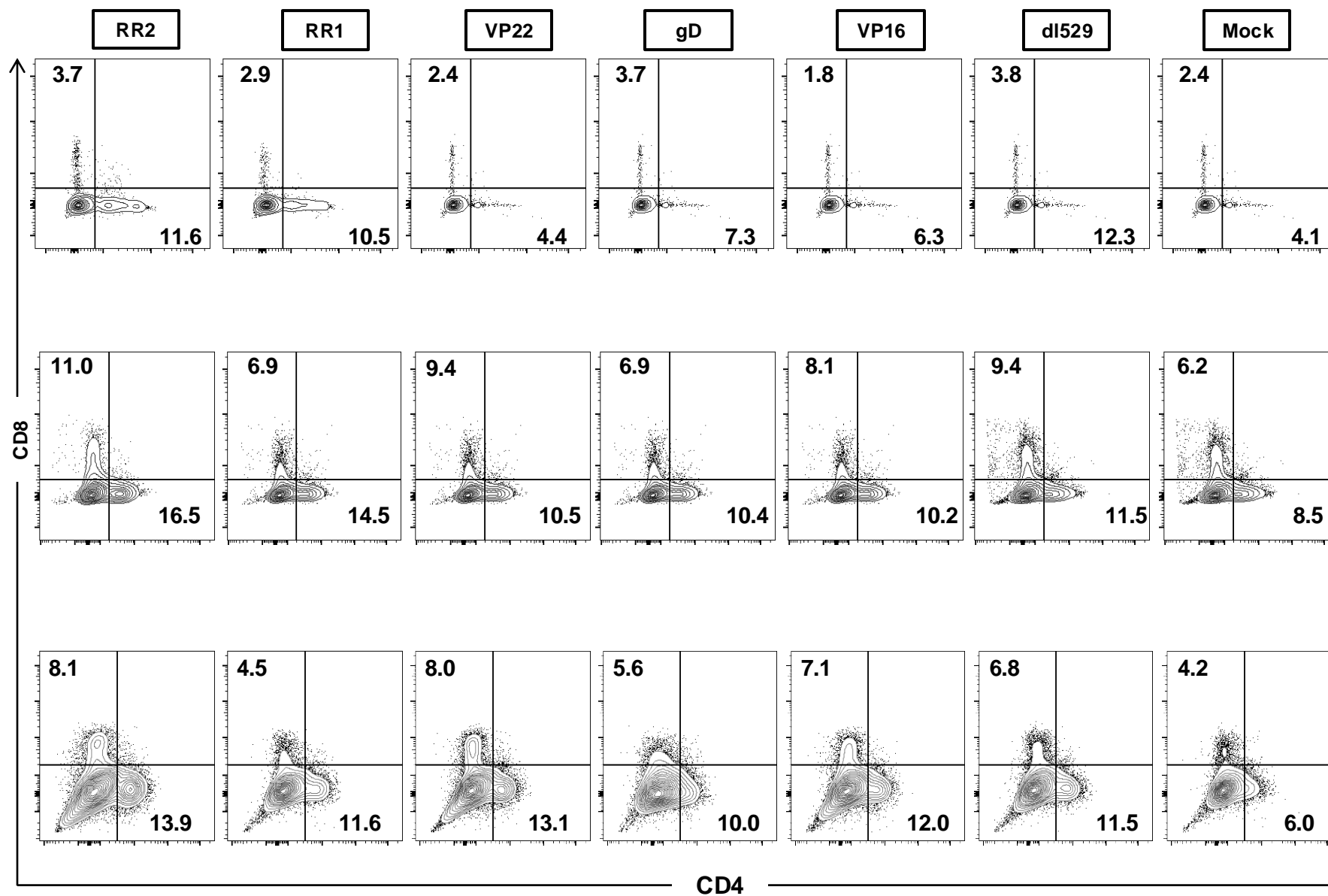

**B**

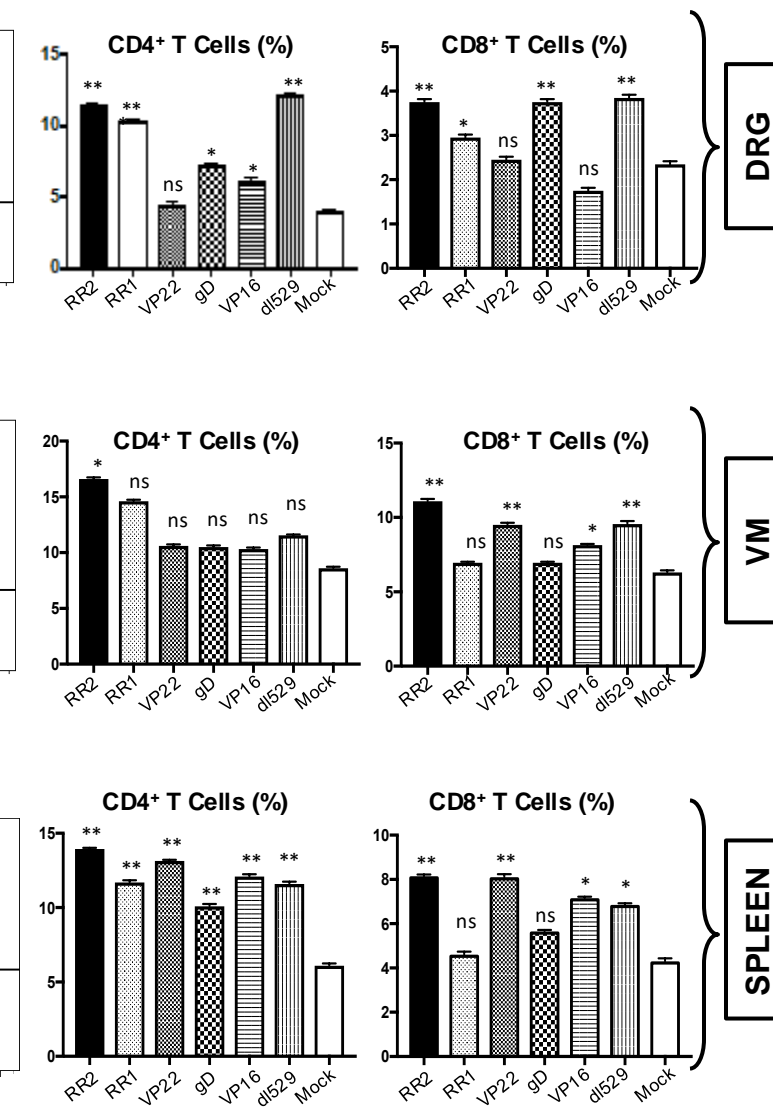

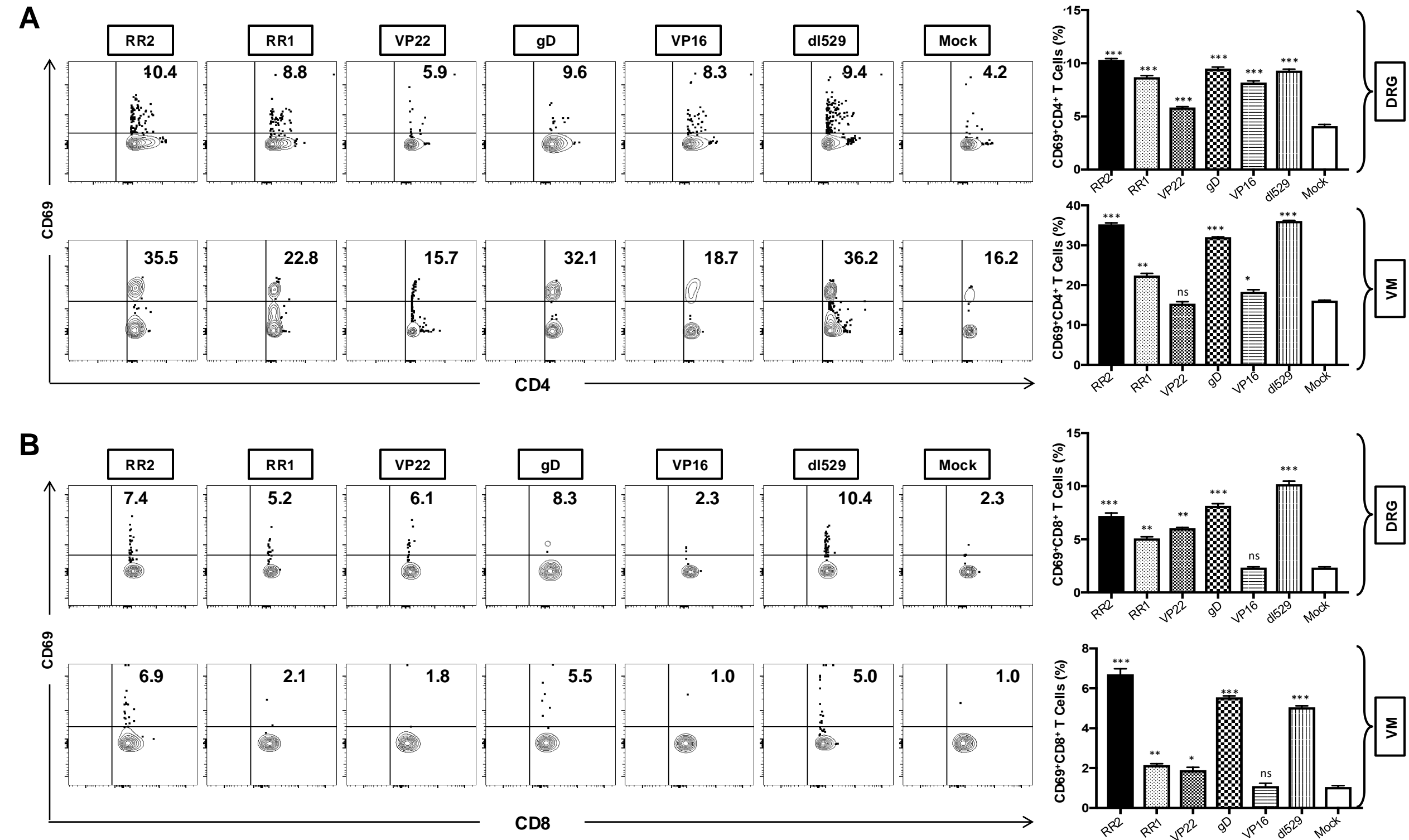

**A**

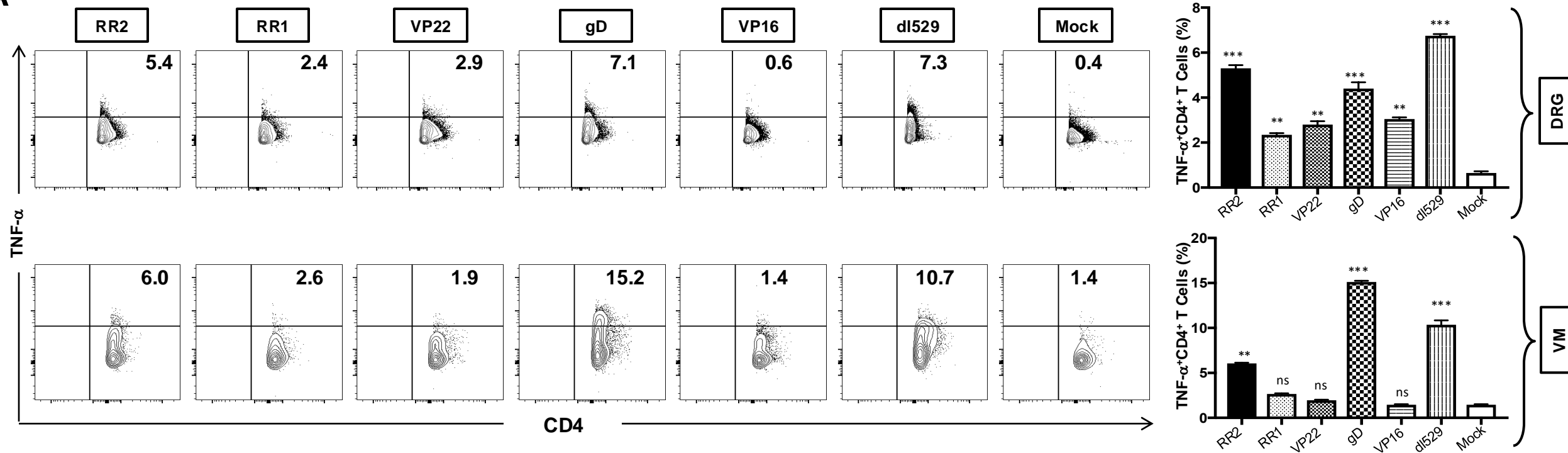

**B**

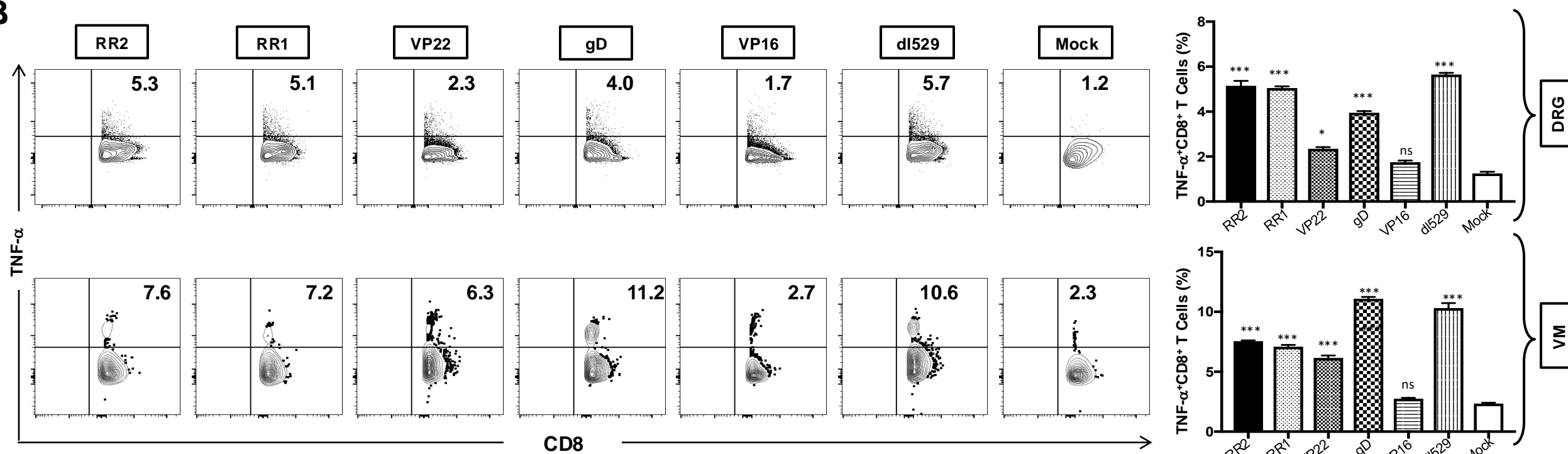

Supplement: Supplementary Figure 1 — Schematic representation of the Ad5 vectors expressing RR1, RR2, VP16, VP22, and gD proteins of HSV-2. [file DataSheet1.pdf]
